# Serum endotrophin relates to blood pressure in young South Asian women but not White women: evidence of ethnic variation in its early vascular implications

**DOI:** 10.1186/s12872-025-05153-1

**Published:** 2025-09-29

**Authors:** Ravi Retnakaran, Dawei Bu, Jiajie Pu, Philip W. Connelly, Anthony J. Hanley, Ningyan Zhang, Zhiqiang An, Bernard Zinman, Philipp E. Scherer

**Affiliations:** 1 Leadership Sinai Centre for Diabetes, University of Toronto, Mount Sinai Hospital, 60 Murray Street, Suite L5-025, Mailbox-21, Toronto, ON M5T 3L9 Canada; 2 Division of Endocrinology, University of Toronto, Toronto, Canada; 3 Lunenfeld-Tanenbaum Research Institute, Mount Sinai Hospital, Toronto, Canada; 4 Touchstone Diabetes Center, The University of Texas Southwestern Medical Center, Dallas, TX USA; 5 Keenan Research Centre for Biomedical Science of St. Michael’s Hospital, Toronto, Canada; 6 Department of Laboratory Medicine and Pathobiology, University of Toronto, Toronto, Canada; 7 Department of Nutritional Sciences, University of Toronto, Toronto, Canada; 8 Texas Therapeutics Institute, Brown Foundation Institute of Molecular Medicine, University of Texas Health Science Center at Houston, Houston, TX USA

**Keywords:** Endotrophin, Cardiovascular, Ethnicity, South Asian, White, Blood pressure

## Abstract

**Background:**

Endotrophin is a novel circulating fibroinflammatory protein that has recently been associated with cardiovascular disease (CVD) and all-cause mortality. However, little is known about its role early in the natural history of vascular disease nor whether it exhibits ethnicity-specific variation. Recognizing that South Asians have an elevated risk of ultimately developing CVD, we sought to address these questions by characterizing endotrophin and its physiologic correlates in young South Asian women as compared to White women.

**Methods:**

From a cohort recruited in pregnancy and followed to 1-year after delivery, we identified 40 participants of South Asian descent and 40 age- and BMI-matched White women. These individuals underwent cardiometabolic characterization at 1-year postpartum, including assessment of anthropometrics, blood pressure, glucose tolerance, insulin resistance, pancreatic beta-cell function, and serum endotrophin.

**Results:**

There was no significant difference in serum endotrophin concentration between South Asians and Whites (median 19.3 ng/ml [interquartile range 14.5–23.8] vs 17.3 ng/ml [14.4–21.7], *p* = 0.37). However, endotrophin was positively correlated with systolic blood pressure (*r* = 0.43, *p* = 0.006) in South Asians but not in White women (*r* = -0.02, *p* = 0.89), mirroring differential associations that were similarly observed with fasting glucose (South Asian: *r* = 0.34, *p* = 0.03; White: *r* = -0.21, *p* = 0.21). Moreover, on multiple linear regression analyses, systolic blood pressure was independently associated with endotrophin in South Asians only (beta = 0.016, *p* = 0.008).

**Conclusion:**

Endotrophin relates to blood pressure in young South Asian women but not White women, suggesting ethnic variation in its early vascular associations that potentially could be relevant to the elevated lifetime risk of CVD in South Asians.

## Introduction

Endotrophin, a circulating peptide derived from the processing of type VI collagen by activated fibroblasts, has been associated with a growing array of conditions in recent years [1]. Initially identified in 2012 [2], it has since been shown to have pro-tumorigenic, pro-fibrotic and pro-inflammatory effects [1, 3]. Over this time, higher circulating endotrophin has been associated with cardiovascular disease (CVD) [4–7], chronic kidney disease [7–9], advanced liver disease [10, 11], chronic obstructive pulmonary disease [12], idiopathic pulmonary fibrosis [13], systemic sclerosis [14], psoriatic arthritis [15], and Crohn’s disease [16] (amongst other conditions), with its fibroinflammatory effects implicated as a potential basis for its emergence across this wide range of conditions [1]. Recently, an individual patient data (IPD) meta-analysis that included 16 cohorts involving 15,205 patients with different non-communicable chronic diseases reported that a two-fold increase in circulating endotrophin predicted an estimated hazard ratio of 2.10 [95% CI: 1.75–2.52] for 3-year all-cause mortality (albeit with heterogeneity between studies) [17]. While the cause of death was not available in many of the cohorts, the authors noted that CVD was likely to be the main cause of mortality [17]. Yoshiji and colleagues recently reported in *Nature Genetics* that endotrophin is a key mediator of the effect of obesity on coronary artery disease [18]. Similarly, recent instrumental variable and colocalization analyses identified endotrophin as causally associated with the risk of coronary artery disease [6], consistent with the demonstration of (i) its presence in atherosclerotic plaque and (ii) the capacity of circulating endotrophin concentration to predict cardiovascular events, cardiovascular death, and all-cause mortality [4]. However, while these data have implicated endotrophin in established CVD, little is known about its role early in the natural history of vascular disease nor whether it exhibits ethnicity-specific variation (i.e. that could have contributed to the heterogeneity in the aforementioned IPD meta-analysis).

In this context, we reasoned that one investigative approach towards addressing these questions would be to evaluate endotrophin in healthy young adults from an ethnic group that has been associated with elevated future risk of CVD. Of note, individuals of South Asian descent have a higher risk of ultimately developing CVD than do White patients [19–22]. Indeed, over median follow-up of 11 years in the United Kingdom Biobank cohort study, individuals of South Asian descent had an adjusted hazard ratio of 2.03 (95%CI 1.86—2.22, *p* < 0.001) for experiencing a CVD event, as compared to those of European ancestry [19]. South Asians also develop CVD at younger age and lower BMI than White individuals [19–22]. Moreover, despite low rates of smoking, South Asian women have disproportionately elevated risk of CVD [20].

Recognizing that pregnancy poses a stress test for many physiologic systems, we posited that South Asian and White women who have recently completed an uncomplicated pregnancy could provide two groups of young healthy participants with a low likelihood of harboring unrecognized underlying comorbidities (that could otherwise confound endotrophin measurements) but with differential future risks of CVD. Specifically, the completion of an uncomplicated pregnancy could serve as a proxy for relative health and the absence of undiagnosed severe medical comorbidity currently. By assessing such women at 1-year postpartum, we reasoned that they would be far enough removed from the pregnancy to limit the impact of gestational changes in physiology but with not so much time having elapsed as to invalidate the proxy of current relative health. Thus, in this study, our objective was to characterize endotrophin and its physiologic correlates in South Asian and White women at 1-year postpartum.

## Methods

This study was performed in the setting of a prospective observational cohort in which women were recruited at the time of antepartum screening for gestational diabetes (GDM) in late 2nd/early 3rd trimester and returned for cardiometabolic characterization at 3-months and 1-year postpartum [23, 24]. The study protocol conforms to the ethical guidelines of the 1975 Declaration of Helsinki and was approved by the Mount Sinai Hospital Research Ethics Board. All women have provided written informed consent for their participation.

### Recruitment of cohort

The protocol for the cohort study has been previously described in detail [23, 24]. In brief, clinical screening for GDM in the obstetrical care of pregnant women at our institution is performed by 50 g glucose challenge test (GCT), whereupon an abnormal response (plasma glucose ≥ 7.8 mmol/L at 1-h after ingestion of 50 g glucose load) leads to referral for a diagnostic oral glucose tolerance test (OGTT). For this cohort study, women were recruited either before or after the screening GCT and all participants then completed a 3-h 100 g OGTT, irrespective of the GCT result (i.e. even if it was normal). As previously described [24], this recruitment strategy was designed to generate a cohort of women comprising the full range of glucose tolerance in pregnancy (from normal to gestational impaired glucose tolerance (GIGT) to GDM) and hence reflecting a broad range of future risk of T2DM and CVD (thereby yielding a setting for studying the early natural history of these conditions) [25–27]. After pregnancy, participants returned to the clinical investigation unit for cardiometabolic characterization at 3-months and 12-months postpartum.

To generate the patient population for the current study, we first identified 40 women who self-identified their ethnic background as South Asian (defined as having ancestry from India, Pakistan, Sri Lanka or Bangladesh). No additional clarification of the participant's definition thereof was obtained*.* They were then matched on both age and BMI at 1-year postpartum with 40 women who self-identified as white. For the current study, we compared these two age- and BMI-matched groups of South Asian and White women at 1-year postpartum.

### Cardiometabolic characterization at 1-year postpartum study visit

The study visit involved interviewer-administered questionnaires, bloodwork, and physical examination, including measurement of weight, waist circumference, and blood pressure. Blood pressure was measured twice 5 min apart by automatic sphygmomanometer (Dinamap Pro 100–400), with the average included in the analyses. Hypertension was defined by (i) average blood pressure ≥ 140/90 mmHg, (ii) known diagnosis of hypertension or (iii) treatment with anti-hypertensive medication. Participants presented for this visit in the morning after overnight fast and underwent a 2-h 75 g OGTT. During the OGTT, venous blood samples were drawn at fasting and at 30-, 60-, and 120-min post-challenge for measurement of glucose and insulin, as previously described [22, 23]. These measurements enabled assessment of glucose tolerance status, insulin resistance, and pancreatic beta-cell function. Glucose tolerance status (normal glucose tolerance, pre-diabetes, diabetes) was defined according to Diabetes Canada clinical practice guidelines, which align with those of the World Health Organization [28]. Area-under-the-glucose-curve (AUC_glucose_) on the OGTT was calculated by trapezoidal rule. Insulin resistance was measured with the Homeostasis Model Assessment of insulin resistance (HOMA-IR) [29]. Beta-cell compensation was assessed by Insulin Secretion-Sensitivity Index-2 (ISSI-2), which is an OGTT-based measure that is analogous to the disposition index obtained from the intravenous glucose tolerance test against which it has been directly validated [30, 31]. Serum endotrophin was measured with an in-house enzyme-linked immunoassay, as previously described [9, 32]. In brief, 96-well plates (Corning Costar) were coated with a monoclonal anti-endotrophin (anti-ETP) specific human IgG antibody prepared in-house at a concentration of 2 μg/mL. Serum samples were titrated at a series of dilutions in 1X PBS and then added to an anti-ETP coated plate. A high affinity specific anti-ETP antibody (ETN-1Rb) was utilized as secondary detection antibody. Anti-Rabbit Fab2-HRP antibody (Jackson ImmunoResearch) was used for detection of endotrophin signals, using the dilution suggested by the manufacturer. A purified endotrophin recombinant protein was titrated in a series of concentrations (0–50 ng/mL) to establish a standard curve for calculation of endotrophin in serum samples. The intra-assay coefficient of variation is 11.2% to 15.6%.

### Statistical analyses

**Fig. 1:**
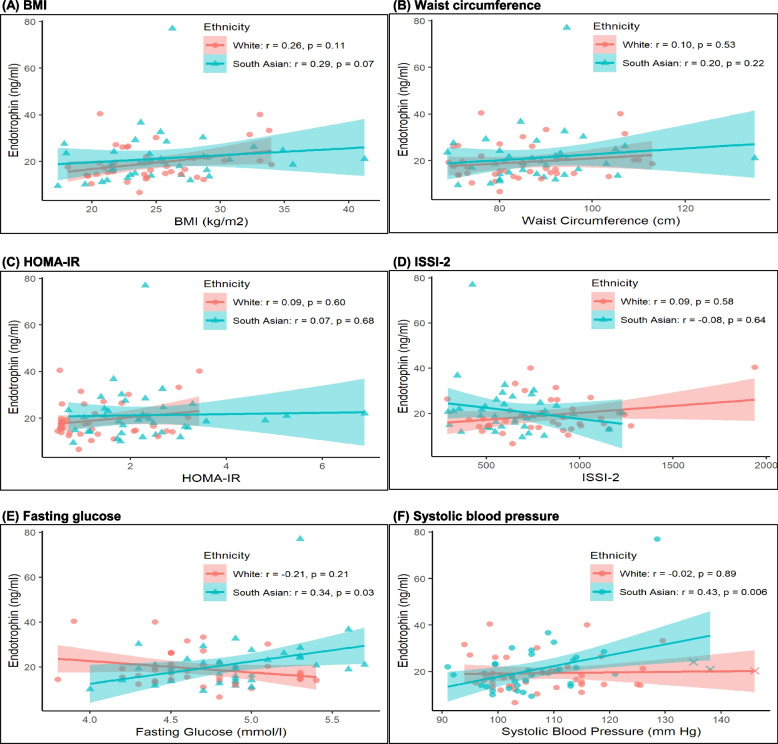
Spearman univariate correlations between endotrophin and (**A**) BMI, (**B**) waist circumference, (**C**) HOMA-IR, (**D**) ISSI-2, (**E**) fasting glucose, and (**F**) systolic blood pressure in White (*n* = 40) and South Asian (*n* = 40) women, respectively. Fitted regression lines with shaded 95% CI are shown. In Panel F,"X"denotes individuals with hypertension

All statistical analyses were performed with R 4.3.0. Continuous variables were tested for normality of distribution, and natural log transformations of skewed variables were used, where necessary, in subsequent analyses. Univariate differences between the South Asian and White groups were assessed by analysis of variance (if normally distributed) or Kruskal–Wallis test (if skewed) for continuous variables and Chi-squared test for categorical variables (Table 1). Backward selection multiple linear regression models of (dependent variable) endotrophin were constructed from the following core set of variables: age, ethnicity, BMI, HOMA-IR, ISSI-2, fasting glucose and systolic blood pressure (Table 2 Model I). Additional models included those in which fasting glucose was replaced with AUC_glucose_ and BMI was replaced with waist circumference (Table 2 Models II-IV). Spearman univariate correlation analyses were performed between endotrophin and cardiometabolic factors (BMI, waist circumference, HOMA-IR, ISSI-2, fasting glucose, and systolic blood pressure) within the South Asian and White groups (Fig. 1). Figure 1 also shows the fitted line from linear regression, with 95% confidence interval. To identify factors independently associated with endotrophin in South Asian and White women, respectively, backward selection multiple linear regression models of (dependent variable) endotrophin were performed within each ethnic group, with the same set of variables as in the full study population in Table 2 (except for ethnicity) and the same approach to the additional models II-IV (Table 3).

**Table 1:** Comparison of age- and BMI-matched White and South Asian women at 1-year postpartum

	**White**	**South Asian**	
**At 1-year Postpartum**	**(*n* = 40)**	**(*n* = 40)**	**P**
Age (years)	35.7 (4.5)	35.2 (4.6)	0.69
Parity:			0.79
1 n(%)	25 (62)	27 (68)	
2 n(%)	12 (30)	11 (28)	
> 2 n(%)	3 (7)	2 (5)	
Glucose tolerance in recent pregnancy:			0.49
GDM n(%)	6 (15)	9 (24)	
GIGT n(%)	9 (23)	10 (26)	
Normal glucose tolerance n(%)	24 (62)	19 (50)	
Current breastfeeding n(%)	17 (45)	15 (41)	0.82
Breastfeeding duration (months)	10.8 (7.1—12.0)	11.0 (6.0—12.0)	0.94
BMI (kg/m^2^)	24.7 (4.4)	25.4 (4.9)	0.48
Waist circumference (cm)	85.0 (12.5)	88.4 (12.1)	0.19
Systolic BP (mm Hg)	108 (11)	108 (10)	0.83
Diastolic BP (mm Hg)	66 (9)	67 (10)	0.48
Hypertension n(%)	1 (2.5)	3 (7.5)	0.99
Insulin resistance:			
HOMA-IR	1.2 (0.6—2.0)	2.2 (1.6—2.7)	**< 0.001**
Beta-cell function:			
ISSI-2	851 (622—971)	620 (500—744)	**0.002**
OGTT:			
Fasting glucose (mmol/l)	4.7 (0.4)	4.9 (0.4)	**0.008**
120-min glucose (mmoll/l)	6.1 (1.8)	6.7 (2.1)	0.18
AUC_glucose_	20.1 (4.4)	22.3 (4.3)	**0.026**
Glucose tolerance:			0.44
Normal glucose tolerance n(%)	35 (88)	30 (75)	
Pre-diabetes n(%)	4 (10)	8 (20)	
Diabetes n(%)	1 (3)	2 (5)	
Endotrophin (ng/ml)	17.3 (14.4—21.7)	19.3 (14.5—23.8)	0.37

Continuous variables are presented as mean (standard deviation), if normal distribution, or as median (interquartile range), if skewed

Bold indicates *p* < 0.05

**Table 2:** Backward selection multiple linear regression models of *(dependent variable)* endotrophin from the following core set of variables: age, ethnicity, BMI, HOMA-IR, ISSI-2, fasting glucose and systolic blood pressure (BP)

Model	Initial Variables	Final Model	Beta	SD	t	p
I	Core set	BMI	0.022	0.009	2.43	0.017
II	Core set with AUC_glucose_ in place of fasting glucose	BMI	0.022	0.009	2.43	0.017
III	Core set with waist in place of BMI	Systolic BP	0.008	0.004	2.03	0.046
IV	Core set with waist in place of BMI and AUC_glucose_ in place of fasting glucose	Systolic BP	0.008	0.004	2.03	0.046

**Table 3:** Backward selection multiple linear regression models of endotrophin in Whites and South Asians, respectively, from the following core set of variables: age, BMI, HOMA-IR, ISSI-2, fasting glucose and systolic blood pressure (BP)

		***Models in Whites***				***Models in South Asians***			
**Model**	**Initial Set of Variables**	**Final Model**	**Beta**	**t**	**p**	**Final Model**^**a**^	**Beta**	**t**	**p**
I	Core set	Log HOMA-IR	0.280	2.36	0.024	Systolic BP	0.016	2.80	0.008
		Fasting glucose	−0.523	−2.66	0.012				
II	Core set with AUC_glucose_	–^b^	–	–	–	Systolic BP	0.016	2.80	0.008
	in place of fasting glucose								
III	Core set with waist	Log HOMA-IR	0.280	2.36	0.024	Systolic BP	0.016	2.80	0.008
	in place of BMI	Fasting glucose	−0.523	−2.66	0.012				
IV	Core set with waist	–^b^	–	–	–	Systolic BP	0.016	2.80	0.008
	in place of BMI and AUC_glucose_								
	in place of fasting glucose								

^a^As the backward selection process in South Asians converged to the same final model each time consisting of only one statistically significant predictor (systolic blood pressure), the estimated coefficient and corresponding *p*-value of systolic blood pressure were also the same each time

^b^No variables remained significant after backward selection

## Results

Table 1 shows the clinical and cardiometabolic characteristics of the 80 study participants, stratified into 2 age- and BMI-matched groups based on their ethnicity, as follows: (i) South Asians (*n* = 40) and (ii) Whites (*n* = 40). There were no differences between the groups in age, parity, glucose tolerance in the recent pregnancy, duration of breastfeeding, BMI, waist circumference, or blood pressure. As anticipated, the women of South Asian origin had higher insulin resistance (HOMA-IR: *p* < 0.001) than White women. The South Asian group also had poorer beta-cell function (ISSI-2: *p* = 0.002). Consistent with these findings, South Asians had higher fasting glucose (albeit in the normal range (mean 4.9 (± 0.4) vs 4.7 (± 0.4) mmol/l, *p* = 0.008)) and higher AUC_glucose_ (*p* = 0.026). There were no significant differences in 2-h glucose or glucose tolerance status. Of note, there was no significant difference in endotrophin concentration between South Asian and White women (median 19.3 ng/ml [interquartile range 14.5–23.8] vs 17.3 ng/ml [14.4–21.7], *p* = 0.37).

We next performed adjusted analyses to determine if ethnicity was independently associated with serum endotrophin. In a series of backward selection multiple linear regression analyses, BMI and systolic blood pressure emerged as the only independent correlates of endotrophin in the 80 study participants (Table 2). Having thus established that endotrophin was not independently associated with South Asian or White ethnicity, we next considered whether its physiologic associations differed between these ethnic groups. On Spearman univariate correlation analyses (Fig. 1), serum endotrophin was not significantly associated with BMI, waist circumference, HOMA-IR or ISSI-2 in either group (Panels A-D). In contrast, endotrophin was positively correlated with fasting glucose in South Asians (Spearman correlation *r* = 0.34, *p* = 0.03; linear regression β = 9.925, 95%CI [1.066, 19.785], *p* = 0.029) but not in Whites (Spearman correlation *r* = −0.21, *p* = 0.21; linear regression β = −5.060, 95%CI [−11.703, 1.582], *p* = 0.13) (Panel E). Moreover, such an ethnicity-specific differential association was even more striking for systolic blood pressure (Panel F), with which endotrophin was positively correlated in South Asians (Spearman correlation *r* = 0.43, *p* = 0.006; linear regression β = 0.465, 95%CI [0.135, 0.794], *p* = 0.007) with no such evidence of correlation in Whites (Spearman correlation *r* = −0.02, *p* = 0.89; linear regression β = 0.024, 95%CI [−0.204, 0.253], *p* = 0.83). On sensitivity analyses adjusted for parity (primiparous versus multiparous), the findings were unchanged with adjusted partial correlations *r* = −0.13 (*p* = 0.44) in the White group and *r* = 0.45 (*p* = 0.0037) in the South Asian group. These findings were also unchanged upon exclusion of the 4 women with hypertension (data not shown).

Having identified differences between South Asian and White women in their univariate correlations of specific physiologic measures with endotrophin, we next performed adjusted analyses with a series of backward selection multiple linear regression analyses within each ethnic group. In White women, backward selection amongst the core set of variables (age, BMI, HOMA-IR, ISSI-2, fasting glucose and systolic blood pressure) identified log HOMA-IR (beta = 0.28, *p* = 0.024) and fasting glucose (beta = −0.523, *p* = 0.012) as independent correlates of endotrophin (Model I). These findings persisted upon the inclusion of waist circumference in place of BMI amongst the variables (Model III) but were eliminated in models wherein AUC_glucose_ replaced fasting glucose (Models II and IV). In contrast, in South Asians, the findings from the same 4 set of models were fully consistent. Specifically, in each model (I-IV), systolic blood pressure emerged as the sole variable independently associated with endotrophin (beta = 0.016, *p* = 0.008), revealing a robust relationship in South Asians that was not present in White women.

## Discussion

In this study, we report 2 main findings. First, there is no significant difference in circulating endotrophin concentration between South Asian and White women at 1-year postpartum. Second, endotrophin is independently associated with systolic blood pressure in South Asian women but not in Whites. It thus emerges that endotrophin exhibits ethnic variation in its early vascular implications that potentially may be relevant to the higher burden of CVD in South Asians.

While endotrophin has been linked to a wide array of conditions, three converging lines of evidence have recently sparked particular interest in its cardiovascular implications. First, amongst its many disease correlates, endotrophin has been most strongly associated with CVD and chronic kidney disease (CKD) [17, 33]. Second, CVD has been implicated as the likely predominant basis by which endotrophin predicts all-cause mortality [17]. Third, endotrophin was recently identified as causally associated with risk of coronary artery disease on instrumental variable and colocalization analyses [6]. Accordingly, we specifically sought to measure endotrophin in young South Asian women at 1-year postpartum, reasoning that their recent uncomplicated pregnancy offered a degree of functional evidence against severe coexisting pathology (which is important when assessing a peptide with so many disease correlates) though this patient population faces an elevated risk of ultimately developing CVD in the future on the basis of its ethnicity.

Studies from North America, Europe and Asia have documented an ~ twofold higher burden of CVD in individuals of South Asian ancestry [19, 34, 35]. While this risk is largely attributed to the respective effects of higher rates of hypertension, diabetes and central adiposity [19, 20, 35], the recognition that these 3 factors do not fully account for the greater burden of CVD in South Asians has spurred investigation into other potential contributors such as social determinants of health, genetic susceptibilities, and non-traditional cardiovascular risk factors [34]. Indeed, relevant to the latter, there exists precedence for ethnic differences in non-traditional risk factors in the observation that South Asians have lower circulating concentrations of adiponectin than White individuals [36]. In a similar vein, the current findings argue against an analogous difference in serum endotrophin concentration at 1-year postpartum but instead identify ethnic variation in the physiologic correlates of this novel fibroinflammatory marker.

Three features support the robustness and plausibility of the ethnicity-specific association between endotrophin and systolic blood pressure that was observed in South Asians. First, the correlation coefficients on the Spearman analyses in Fig. 1F suggested absolutely no association in Whites (*r* = −0.02), in stark contrast to that in South Asians (*r* = 0.43, *p* = 0.006) in age- and BMI-matched groups of equal size. Second, on the ethnicity-restricted multiple linear regression analyses (Table 3), systolic blood pressure consistently emerged as the sole variable independently associated with endotrophin in South Asians in all 4 models. Third, this difference between South Asian and White women was evident in the setting of both groups exhibiting normal blood pressure measurements, with very low rates of hypertension (Table 1), suggestive of ethnicity-specific differential biology. The biologic basis for these findings is speculative at this time but theoretically could include a mechanism whereby the fibrotic or inflammatory effects of endotrophin predominantly impact determinants of blood pressure (such as vascular tone or endothelial function) in young South Asians but not in White women. Conversely, in White women, there was a modest inverse association between fasting glucose and endotrophin, though this signal was not as robust. Taken together, these data are raising the possibility of ethnic differences in the physiological action profile of endotrophin. While the underlying basis for such differences cannot be currently ascertained, possibilities could theoretically include ethnicity-specific genetic polymorphisms that impact endotrophin signaling pathways or differential effects of diet and lifestyle that might direct endotrophin activity towards specific target tissues (such as vasculature or adipose tissue). The myriad possibilities at play need further study and, given their potential complexity, might benefit from the application of artificial intelligence methodologies [37].

An important limitation is that the cross-sectional design precludes inference on causality in the association between endotrophin and systolic blood pressure in South Asian women. It remains to be determined whether one factor influences the other or if there exists an indirect relationship wherein both are affected by a third factor. Another limitation is that, in the setting of the observational study design, unmeasured confounders can potentially influence observed associations. Such factors could include socioeconomic status, dietary factors, physical activity, levels of acculturation or inflammatory factors. The generalizability of the study population also remains to be determined since we cannot fully exclude the possibility of an effect of the postpartum status of the women. Indeed, we specifically did not measure endotrophin at the 3-month postpartum visit because of the possibility that residual effects of the physiologic changes associated with gestation could impact findings. For this reason, endotrophin was measured at 1-year after delivery and it is reassuring that there were no differences between the groups in gestational glucose tolerance, parity or duration of breastfeeding (Table 1). An additional factor relevant to generalizability is that both White and South Asian ethnicity were self-reported. While South Asian ethnicity was clarified with participants as reflecting ancestry from India, Pakistan, Sri Lanka or Bangladesh, it should be recognized that these populations are heterogenous with distinct cultural and genetic backgrounds. With self-identified White ethnicity, no additional clarification of the participant’s definition thereof was obtained, raising the possibility of heterogeneity in this group as well.

Another limitation is that the sample size was relatively modest, though it is encouraging that post-hoc power calculation revealed ~ 86% power to detect the observed correlation between endotrophin and systolic blood pressure in South Asians with two-sided test. Due to the modest size of the ethnicity-specific subgroups (*n* = 40), we deliberately applied a backward selection regression method starting with 6 candidate predictors to reduce model complexity and to exclude those with minimal explanatory value. In South Asians, the 4 backward selection models presented in Table 3 consistently converged to the same final model with only one significant predictor, suggesting some model stability. However, it should be recognized that this data-driven selection process with modest sample size increases the risk of overfitting and selection bias, such that the study should be interpreted as exploratory, with findings interpreted accordingly. Moreover, reference ranges and clinically validated thresholds for endotrophin have not been established. Noting the intra-assay coefficient of variance (11.2% to 15.6%) for the current research assay, it would be helpful to replicate the analyses with a validated commercially-available assay but none is yet available (owing to the relative novelty of endotrophin). The absence of a commercial assay at this time precludes any immediate application but underscores the need for further study.

The current findings also hold additional research implications. First, future clinical studies now need to consider the possibility of ethnic variation in endotrophin biology, whether in the circulating concentrations of this fibroinflammatory protein or in its physiologic associations (such as with inflammatory markers or mediators). Second, longitudinal studies with measurement of endotrophin early in the natural history of CVD are needed to address its potential contribution to the pathophysiology of vascular disease over time. In particular, its relation with longitudinal changes in blood pressure warrant study. Third, the association between endotrophin and systolic blood pressure in young normotensive South Asian women raises the possibility that differences in circulating endotrophin concentration may ultimately emerge over time as they grow older. Indeed, these data identify endotrophin as a potential contributor to the elevated cardiometabolic risk of South Asians that warrants further longitudinal evaluation for changes in both circulating concentration and pathophysiologic correlates/outcomes. Finally, while sex differences in endotrophin biology have not been noted to date, the current findings underscore the need for consideration of this possibility in future studies.

In conclusion, at 1-year postpartum, there is no significant difference in serum endotrophin concentration between age- and BMI-matched South Asian and White women, either before or after covariate adjustment. However, there exists an independent association between endotrophin and systolic blood pressure in young South Asian women that is not present in their White peers, though causality cannot be inferred. These data provide evidence of ethnic variation in the early vascular implications of endotrophin that warrants further study in the context of the elevated burden of CVD in South Asians.

## Data Availability

De-identified data can be made available under restricted access from the corresponding author, for academic purposes, subject to a material transfer agreement and approval of the Mount Sinai Hospital Research Ethics Board.

## Abbreviations

AUC_glucose_: Area-under-the-glucose-curve
CVD: Cardiovascular disease
HOMA-IR: Homeostasis Model Assessment of Insulin Resistance
IPD: Individual patient data
ISSI-2: Insulin Secretion-Sensitivity Index-2
OGTT: Oral glucose tolerance test
T2DM: Type 2 diabetes
